# Functional Stability of Plasminogen Activator Inhibitor-1

**DOI:** 10.1155/2014/858293

**Published:** 2014-10-15

**Authors:** Songul Yasar Yildiz, Pinar Kuru, Ebru Toksoy Oner, Mehmet Agirbasli

**Affiliations:** ^1^Department of Bioengineering, Marmara University, 34722 Istanbul, Turkey; ^2^Department of Cardiology, Faculty of Medicine, Marmara University, Selimiye M. Tibbiye C. No. 38, Haydarpasa, 34668 Istanbul, Turkey

## Abstract

Plasminogen activator inhibitor-1 (PAI-1) is the main inhibitor of plasminogen activators, such as tissue-type plasminogen activator (t-PA) and urokinase-type plasminogen activator (u-PA), and a major regulator of the fibrinolytic system. PAI-1 plays a pivotal role in acute thrombotic events such as deep vein thrombosis (DVT) and myocardial infarction (MI). The biological effects of PAI-1 extend far beyond thrombosis including its critical role in fibrotic disorders, atherosclerosis, renal and pulmonary fibrosis, type-2 diabetes, and cancer. The conversion of PAI-1 from the active to the latent conformation appears to be unique among serpins in that it occurs spontaneously at a relatively rapid rate. Latency transition is believed to represent a regulatory mechanism, reducing the risk of thrombosis from a prolonged antifibrinolytic action of PAI-1. Thus, relying solely on plasma concentrations of PAI-1 without assessing its function may be misleading in interpreting the role of PAI-1 in many complex diseases. Environmental conditions, interaction with other proteins, mutations, and glycosylation are the main factors that have a significant impact on the stability of the PAI-1 structure. This review provides an overview on the current knowledge on PAI-1 especially importance of PAI-1 level and stability and highlights the potential use of PAI-1 inhibitors for treating cardiovascular disease.

## 1. Introduction

Plasminogen activator inhibitor-1 (PAI-1) is a member of serine proteinase inhibitors (serpin) superfamily. Each serpin consists of about 350–400 amino acid residues (depending on the degree of glycosylation) with molecular masses in the range of 38 to 70 kDa [1]. “Stressed-to-relaxed” conformational change is the distinguishing feature of the serpin protein family members that leads to considerable thermodynamic stabilization and inhibitory mechanism of serpins is based on this transition. Serpins are divided into two groups, that is, the inhibitory serpins and the noninhibitory serpins [2]. PAI-1 belongs to the inhibitory serpins group, that is, the inhibitor of plasminogen activators. Two types of PAI-1, tissue-type plasminogen activator (t-PA) and urokinase-type plasminogen activator (u-PA), are reported [3]. Both types of plasminogen activators are members of serine proteases. Circulating proenzyme plasminogen is cleaved via these serine proteases, which forms the active protease plasmin.

**Figure 1:**
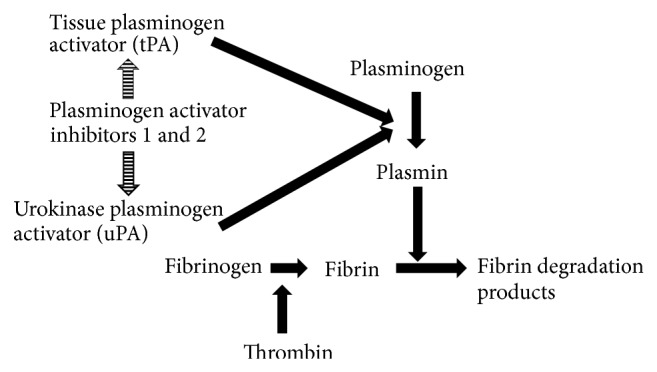
Role of PAI-1 in fibrinolytic system.

Lysis of fibrin in a blood clot and pericellular proteolysis are the results of activation of plasminogen by t-PA and u-PA, respectively. As potential check points in the regulation of fibrinolysis, the activity of plasmin can be directly inhibited by *α*2-antiplasmin or thrombin activatable fibrinolysis inhibitor (TAFI) or plasminogen activator inhibitors can block the conversion of plasminogen to plasmin [4]. PAI-1 is the most important direct physiological inhibitor of t-PA and u-PA and major regulator of the fibrinolytic system (Figure 1) [5]. Severity and unfavorable outcomes were reported in a number of diseases due to elevated plasma levels of PAI-1 antigen [6–9] and activity [9–11]; as a result, PAI-1 is considered as a biomarker and potential molecular target for therapeutics.

## 2. Biochemistry of PAI-1

PAI-1 is a 47 kD single chain glycoprotein consisting of 379 or 381 amino acids depending on heterogeneity of N-terminal caused by two possible cleavage sites for signal peptidases [12] and there is no cysteine in the PAI-1 molecule. PAI-1 is a globular protein with 3 beta-sheets (A, B, and C) and 9 alpha-helices (hA–hI) [13]. An exposed peptide loop of PAI-1 reactive centre loop (RCL) has significant importance for the inhibitory mechanism [14].

Purification of human PAI-1 to apparent homogeneity and monoclonal antibodies against human PAI-1 were reported in 1984 [15] and 1986 [16, 17], respectively. The isolation of full length cDNA encoding human PAI-1 was described independently by four groups in 1986 (NCBI accession number M16006) [18–21].

The cDNAs encoding PAI-1 from rat [22], bovine [23], mouse [24], rabbit [25], porcine [26], and vervet monkey [27] have been cloned and these studies revealed 81 to 97% nucleic acid identity and 78 to 97% amino acid identity with human PAI-1 [28].

**Figure 2:**
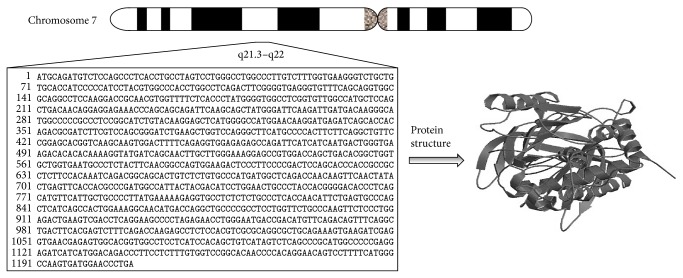
Genetic loci and protein structure of PAI-1.

The human PAI-1 gene is located on chromosome 7 (q21.3–q22) (Figure 2), spans approximately 12200 base pairs, and consists of nine exons and eight introns [29]. Several groups have analyzed and sequenced the whole PAI-1 gene [29–33]. Several agents including dexamethasone, endotoxin, lipopolysaccharide, growth factor, thrombin, interleukin-1, tumor necrosis factor, insulin, very low-density lipoprotein (VLDL), low-density lipoprotein (LDL), and lipoprotein stimulate the synthesis and secretion of PAI-1 [34, 35].

Several genetic variations have been described in the PAI-1 gene. PAI-1 polymorphisms include two dinucleotide (CA) repeats in the promoter and in the intron 4, a Hind III restriction fragment length polymorphism (RFLP) and an insertion (5G)/deletion (4G) polymorphism at position −675 of the PAI-1 promoter, a T-to-G substitution at position +11 053, two G-to-A substitutions at positions −844 and +9785 in the intron 7, a deletion of nine nucleotides from a threefold repeated sequence between nucleotides +11 320 and +11 345, and a G +12 078 A polymorphism in the 3′ untranslated region [36]. Homozygosity for 4G in the 4G/5G polymorphism has been correlated with elevated PAI-1 protein levels, increased risk of thrombosis, and impaired fibrinolysis [37].

## 3. Physiological Function of PAI-1

PAI-1 can be synthesized in various tissues and cell types such as liver, spleen, adipocytes, hepatocytes, platelets, megakaryocytes, macrophages, smooth muscle cells, placenta, and endothelial cells [38]. PAI-1 forms a covalent bond with both t-PA and u-PA (1 : 1 molar ratio) and blocks degradation of active fibrin by preventing the generation of plasmin (protease of fibrinolysis) from the inactive precursor plasminogen [39, 40]. PAI-1 has a significant role in acute thrombotic events such as DVT and MI, type-2 diabetes, cancer, and fibrotic disorders including atherosclerosis and renal and pulmonary fibrosis [39–41].

Tissue plasminogen activator (t-PA) and plasminogen activator inhibitor-1 (PAI-1) directly influence thrombus formation and degradation and thus risk for arterial thrombosis. PAI-1 is a procoagulant, proinflammatory, and profibrotic molecule [39, 40]. PAI-1 is frequently expressed in injured tissues including myocardium and brain and the PAI-1/tissue plasminogen activator (tPA) ratio is indicative of a patient's fibrinolytic balance which can indicate thrombus and stroke risk. Studies indicate evidence for the endothelial activation in small vessel brain injury, associated with low levels of PAI-1. Prior studies associate high levels of t-PA and differences in activity of components of the fibrinolytic system with white matter lesion development [42].

Atherosclerosis is an inflammatory process that results in lipid accumulation at arterial wall. Adaptive and innate immunity have active roles in atherosclerotic process. Mainly, monocytes give rise to macrophages and they become foam cells in the arterial intima, which is the hallmark of the arterial fatty streak. Mast cells, platelets, and T and B cells also play role in atherogenesis [43]. The major event behind atherosclerosis is inhibition of fibrinolysis due to increased plasminogen activator inhibitor-1 (PAI-1) levels, indicator of ineffective fibrinolysis. This leads to increased thrombus formation [44] and makes the plaque unstable. High serum glucose concentration also has been shown to be associated with elevated PAI-1 levels. Increased PAI-1 levels have been encountered in many disease conditions, including metabolic syndrome [45], diabetes [44], and obesity. Recent studies showed that PAI-1 also has role in adipose tissue development and in the control of insulin signaling in adipocytes [45].

Thrombolytics are the major agents in the management of acute thrombotic vascular events. PAI-1 is the primary inhibitor of both tissue-type and urokinase-type plasminogen activators, which inhibits fibrinolysis and has causal relationship with various vascular complications [40]. Additionally, PAI-1 also has pivotal role in the innate immunity by regulating cell migration and phagocytosis. PAI-1 stimulated the migration of monocytes and macrophages by interacting with LRP or tPA. PAI-1 also promoted the migration of lymphocytes and neutrophils into inflammatory sites [46]. PAI-1 is spontaneously converted into a thermodynamically stable latent form and has a short half-life (*t*_1/2_) of around 2 h [47]. Plasminogen activators (PA) and PA inhibitors (PAI) are balanced in blood and regulate the conversion of plasminogen to plasmin [40]. Plasmin exerts proteolytic activity on a wide range of proteins including matrix metalloproteinases (MMPs), transforming growth factor (TGF-b1), laminin, type IV collagen, and fibronectin. Plasma concentration of PAI-1 antigen and activity levels of PAI-1 exert prominent differences even in normal populations [48].

Through the regulation of the urokinase-type and tissue-type plasminogen activators, PAI-1 takes role in such physiological processes as wound healing and tissue remodeling. This key role gives great importance to PAI-1 in many pathophysiological conditions including cardiovascular diseases and cancer metastasis and spread [1, 49].

PAI-1 deficiency in experimental animal models was associated with protective effects against L-NAME-induced perivascular fibrosis, kidney fibrosis, and bleomycin-induced lung fibrosis [50, 51].

## 4. Clinical Implications of PAI-1

PAI-1 is principal inhibitor of plasminogen activation and, thus, has been of particular focus in cardiovascular disease. Studies display a strong correlation between serum PAI-1 levels and cardiovascular risk in different clinical settings. For instance, elevated serum PAI-1 is associated with risk for myocardial infarction (MI), recurrent MI, angina pectoris, and atherosclerosis [52–56]. In addition to its modulatory role in the fibrinolytic system, plasminogen activator inhibitor-1 (PAI-1) is a well-characterized regulator of matrix remodeling. Experimental and clinical evidences suggest that PAI-1 not only is a biomarker but also is in fact a pivotal mediator of vascular disease, cancer, asthma, insulin resistance, and obesity [57–59]. The native plasminogen activator inhibitor-1 (PAI-1) represents an active conformation that spontaneously converts to an inactive latent form. PAI-1 has a short span of activity with a half-life (*t*_1/2_) around 2 hours followed by spontaneous conversion into a latent form [60, 61]. Enhanced stability of PAI-1 is associated with biological changes across multiple systems. Transgenic expression of a conformationally stabilized active human PAI-1 is associated with a number of phenotypic abnormalities including age-dependent spontaneous coronary arterial thrombosis and alopecia areata [62]. We observed markedly enhanced functional stability of PAI-1 in patients with a rare thrombotic skin condition: livedoid vasculopathy [41]. Similarly, we reported nearly 50-fold increase in stability of PAI-1 in a family with extensive cardiovascular disease and vitiligo [47]. Sequencing of PAI-1 gene was performed in three subjects with the highest functional stability levels; however, results did not display any discerning alterations in the gene sequence [47]. While the exact mechanism of increased stability of PAI-1 activity is not known, it may be due to posttranslational modifications or increased binding affinity for a stabilizing cofactor. Thus, these findings suggest that the increased stability of PAI-1 activity may contribute to the commonality across multiple systems and disease phenotypes.

## 5. Conformational Structure of PAI-1 

PAI-1 has three interconvertible conformations: active, latent, and substrate forms [63, 64]. The first structure of a serpin (cleaved *α*1-antitrypsin) was solved in 1984 [65]. In 1992, Mottonen et al. [66] characterized the first PAI-1 (latent) conformation structurally. Subsequently, two other groups clarified a structure of PAI-1 in the latent conformation [67]. Since the exposed RCL is not in its most stable conformation, native forms of serpins are called “metastable,” that is, the unique feature of serpin family. The mobility of the RCL guides the inhibitory activity of serpins, and the RCL becomes inserted easily into the central *β*-sheet upon cleavage by proteases and formation of a covalent acyl-intermediate. The driving force for this conformational change is thermodynamic, yielding a more stable and more extensive central *β*-sheet.

At first, interaction between serpin and protease gives rise to a noncovalent Michaelis complex formation in which the P1–P1′ bond in the RCL docks into the active site. Then, the cleavage of P1–P1′ bond causes covalent linkage of P1 residue to the active site serine of the protease by an ester bond. After that, the N-terminal residues of the RCL becomes inserted into *β*-sheet A, whereas translocation of the protease occurs to the opposite pole of the serpin and trapped in an inactive form with a distorted active site [68]. Serpins have common motifs that are also shared by the tertiary structure of active PAI-1. One of the motifs that is shared is the solvent-exposed RCL of about 20 amino acids long (designated P16 through P80, including the bait peptide bond Arg^346^–Met^347^ (P1–P10)) [66]. In its active conformation, protease reacts with P1–P10 reactive center bond and cleaves this bond; then, the amino terminal part of the RCL has inserted RCL into *β*-sheet A; as a result, the protease has been relocated to the opposite pole of PAI-1 [69]. Finally, a 1 : 1 SDS-stable acyl-enzyme complex has been formed [70]. The protease is inhibited as a consequence of distortion of the active site of the protease during this remarkable conformational change [68]. In the case of hampering of RCL insertion through cleavage in one way or another, release of PAI-1 from the protease is more likely and hence PAI-1 acts only as a substrate of the protease and not as an inhibitor [63, 69]. Mutations in the hinge region [60, 71, 72] as well as changes in external conditions [2, 73–76] or the addition of monoclonal antibodies [77–81] can induce substrate behavior of PAI-1.

## 6. Importance of PAI-1 Level

The normal plasma concentration of PAI-1 antigen is considered to be between 6 and 80 ng/mL. One unit of PAI-1 activity is defined as the level that can neutralize one unit of single chain tPA in 10 minutes. Activity levels range from 0 to 50 U/mL with antigen levels from few to 100 ng/mL [48].

High PAI-1 levels are associated with an increased cardiovascular risk of atherothrombosis, dyslipidemia, hyperinsulinemia, and hypertension [82, 83].

In 1989, presence of significant correlation of PAI-1 levels with both total cholesterol and total triglycerides was reported [84]. One year later, similar results were obtained by another group [85]. These results suggest that hypertriglyceridemia may be associated with increased levels of PAI-1.

Research group of Madan et al. [86] compared type-2 diabetic patients with and without microvascular complications. Increased levels of PAI-1 were found in patients with microvascular complications. It is reported that PAI-1 levels lead to the procoagulant state found in diabetes. Contribution of this state to the major vessel diseases and microvascular complications is also significant findings of the study [86].

In the study of Adly et al. [87], PAI-1 levels in children and adolescents with type-1 diabetes were determined and relation between PAI-1 levels and some risk factors such as glycemic control, microvascular complications, and carotid intima-media thickness (CIMT) for the development of atherosclerosis was investigated. Significant difference between patients with and without microvascular complications was found. Higher PAI-1 levels are detected in patients with microvascular complications, microalbuminuria, or peripheral neuropathy. Results of this study support the potential usefulness of PAI-1 in early detection of risk of vascular complications.

It is reported that increased PAI-1 expression triggers signaling pathways that alter tumor microenvironment and inhibit apoptosis and promotion of angiogenesis which enhance tumor growth [88]. Moreover, Knudsen et al. [89] reported correlation of elevated levels of PAI-1 with HIV-1-infected patients. In this respect, Ferroni et al. [90] investigated elevated plasma PAI-1 levels as a prognostic indicator of breast cancer. Results of this study indicate that elevated plasma PAI-1 levels were associated with increasing tumor stage and disease relapse, which encourage future investigations addressing the role of plasma PAI-1 levels in the management of patients with breast cancer and in providing the rationale for new therapeutic strategies.

While numerous investigations have reported increased levels of PAI-1 and its physiological function, reports of PAI-1 deficiency are more limited. The first case was published in 1989 and reported the correlation between low levels of PAI-1 and lifelong bleeding disorder [91]. Two years later, a 36-year-old patient with undetectable plasminogen activator inhibitor type-1 (PAI-1) antigen and activity was reported. This report indicated association of a severe deficiency of PAI-1 with a delayed type bleeding tendency and revealed the importance of plasma PAI-1 for the stabilization of the hemostatic plug [92]. In 1992, homozygous frame-shift mutation within the PAI-1 gene that results in the formation of a premature stop codon has been identified for the first time. This report provides opportunity to assess its function* in vivo* because this molecular defect results in complete loss of expression of human PAI-1. Results indicated that PAI-1 functions* in vivo* to regulate hemostasis and take role in abnormal bleeding and this study has accelerated further studies on PAI-1 deficiency [93].

Afterwards, many studies about the correlation between PAI-1 deficiency and bleeding diathesis have been reported and specific genetic mutation associated with PAI-1 deficiency has been published [94–103]. Mild to moderate bleeding disorders are caused by PAI-1 deficiency. Incidence of PAI-1 deficiency is quite rare since the lack of a sensitive PAI-1 activity assay obstructs diagnosis of this condition.

## 7. Functional Stability of PAI-1

When PAI-1 is synthesized in endothelial cells and released into blood, it is in a functionally active form [104], which is the native conformation, and has the inhibitory activity towards its target proteases. Among serpins, active conformation of the PAI-1 is the least stable. Spontaneous activity loss of active form of PAI-1 with a functional half-life of 1-2 h at 37°C under normal conditions has been reported [61]. Interaction with the target proteases is not in use in the nonreactive latent form of PAI-1. Partial reactivation of the latent form can be achieved by denaturing agents and subsequent refolding [105], and also* in vivo* reactivation of latent PAI-1 has been observed [106]. The conversion of PAI-1 from the active to the latent conformation appears to be unique among serpins in that it occurs spontaneously at a relatively rapid rate [107, 108]. It is believed that latency transition represents a regulatory mechanism that reduces the possibility of thrombosis from a prolonged antifibrinolytic action of PAI-1 [14].

*In vitro*, by the movement of the RCL into the central *β*-sheet, the conformation of PAI-1 spontaneously converts from the active state to an energetically more favorable inactive latent state [66, 108, 109].

One of the major structural rearrangements identified for a folded protein is PAI-1 latency transition that occurs without a concomitant change in covalent structure; even so, how the sequence of conformational changes occurs through latency transition remains largely unknown [110].

In order to increase the stability of active PAI-1, many studies have been done. Eren et al. reported a number of phenotypic abnormalities including age-dependent spontaneous coronary arterial thrombosis and alopecia areata in a transgenic expression animal model of conformationally stabilized active human PAI-1 [62].

Increased stability of PAI-1 activity may contribute to the pathophysiology of several complex diseases. It was recently observed that the functional stability of PAI-1 was markedly increased (nearly 43-fold) in plasma from the patients with thrombotic skin disorder such as livedoid vasculopathy. The mechanism of enhancing functional stability of PAI-1 activity is unknown.

It has been shown that environmental conditions or interaction with other proteins can have a considerable impact on the stability of the PAI-1 structure. Besides, construction of a variety of mutants has been reported in order to prolong the half-life of PAI-1.

### 7.1. Environmental Conditions

Stable active PAI-1 structure was reported in the conditions of a low pH (≈5.5), a high salt concentration (1 M NaCl), and a low temperature (4°C) [111, 112].

The stability of PAI-1 is pH-dependent and it is reported that PAI-1 is more stable at pH 5.5 and 37°C; it has a half-life of about 16 h [111]. It is thought that one or several histidine residues contribute to the acid stabilization of PAI-1 because a decrease in pH is accompanied by a protonation of imidazole groups [113]. In 2000, it was demonstrated that His^364^ is responsible for the pH-dependent stability of PAI-1 [114]. Strong interactions between electronegative ions and the partially positive nitrogen atoms of the anion binding site increase the energy barrier for the active to latent transition [67]. Additionally, 15-fold stability increase has been reported in the case of arginine binding [115].

PAI-1 can be partially reactivated to the active form if it is exposed to the high concentration of certain denaturants, including guanidinium chloride and urea, after the refolding in a slightly acidic dialysis buffer [105, 111, 116]. It was also reported that phospholipids might convert latent PAI-1 to the active form [117] and PAI-1 synthesized in bovine endothelial cells could be reactivated by heating it to 100°C [118].

### 7.2. *In Vivo* Stabilization

Vitronectin is a multifunctional glycoprotein found in blood and in the extracellular matrix and it can bind collagen, plasminogen, glycosaminoglycans, and the urokinase-receptor. It stabilizes the inhibitory conformation of PAI-1 [119], decreasing its rate of spontaneous inactivation [120, 121].

Plasma binding protein vitronectin stabilizes the PAI-1 molecule at least two to threefold by binding to it [67, 114]. PAI-1 and vitronectin are believed to be colocalized in the extracellular matrix [121, 122]. Half-life of PAI-1 is about 2 h at 37°C and neutral pH in the absence of vitronectin, but twofold increase in the half-life has been reported in the presence of vitronectin [123]. *α*1-acid glycoprotein is another ligand that can stabilize the PAI-1 activity. However, the stabilizing effect of *α*1-acid glycoprotein is not pronounced as well as vitronectin [124].

### 7.3. Mutagenesis

Several studies on PAI-1 have been reported where either site-directed mutagenesis or random mutagenesis has increased its functional stability [60, 61, 73]. It is reported that random mutagenesis of large number of residues in different parts of the PAI-1 gives rise to clones with increased stability of PAI-1.

Using a random mutagenesis approach, Berkenpas et al. [61] identified a set of mutations that had considerable stabilizing effects on the PAI-1 stability. Exceptional stability was displayed by few single mutations and, significantly, 9-fold stabilization of the PAI-1 activity (*t*_1/2_ ≈ 18 hours) was detected in the mutation of the isoleucine residue to a leucine at position 91. On the other hand, combination of several changes gives rise to about 150 h half-life. However, several changes in combination give rise to PAI-1 molecule with a half-life of about 150 h [61]. The most stable variant they identified was a quadruple mutant (N150H, K154T, Q319L, and M354I) with a half-life of approximately 145 hours at 37°C, with a 72-fold stabilization in comparison to human PAI-1 wild-type.

A few years later, addition of a fifth mutation, Q301P, to this quadruple mutant was reported by Vleugels et al. [125] and very similar properties were observed in both cases.

It is reported that the mutations at positions 154, 319, and 354 contribute the most to the stabilization in these stable variants [125, 126]. In further studies, it has been demonstrated that the combination of mutations at positions 50, 56, 61, 70, 94, 150, 222, 223, 264, and 331 increased the half-life of PAI-1 245-fold [127] and a disulfide mutant with a more than 350-fold increased stability was reported in 2003 [128]. Results are summarized in Table 1.

### 7.4. Glycosylation

Glycosylation pattern analysis of natural human PAI-1 showed that different glycosylation patterns are present in different cell sources that support presence of tissue-specific glycosylation pattern [129].

Most biochemical and structural studies have been performed with nonglycosylated PAI-1 produced in* Escherichia coli*. However, in natural cell lines and eukaryotic cells, glycosylated PAI-1 is present. Although biochemical properties of proteins are influenced significantly by glycosylation, minor differences were reported for nonglycosylated and glycosylated PAI-1 [76, 130, 131]. Three potential N-glycosylation sites, Asn^209^, Asn^265^, and Asn^329^, have been identified in human PAI-1 [132, 133].

It was demonstrated that human PAI-1 displays a heterogeneous glycosylation pattern of asparagines Asn^209^ and Asn^265^, while Asn^329^ is not utilized [133]. The latent transition of nonglycosylated PAI-1 was more easily enhanced by a nonionic detergent when compared with glycosylated PAI-1 [134].

It is confirmed that only two N-linked glycosylation sites are actually used when glycosylation pattern of natural human PAI-1 was analysed in different cell sources. However, heterogeneous, tissue-specific glycosylation pattern was also observed [129]. It is hypothesized that PAI-1 circulates in both glycosylated and nonglycosylated states* in vivo*, depending on the cell type that expresses the protein [129, 135]. Despite the fact that glycosylation of PAI-1 is not a prerequisite for its activity [130], glycosylation status of PAI-1 is critically significant in the development of PAI-1 inhibitors [12, 28]. Research of van de Craen et al. [12] indicated that targeting glycosylated PAI-1 can be efficient therapeutic approach to control PAI-1 levels* in vivo*.

Bager et al. found that single glycosylation site is present in PAI-1 from bony fish. In the same study, recombinant PAI-1 of zebrafish (*Danio rerio*) PAI-1 (zfPAI-1) was produced [136].

Interestingly, slow latency transition was detected in a zfPAI-1 produced in a glycosylated form, whereas rapid conversion to latent state was observed in nonglycosylated zfPAI-1. This effect can be explained by simple steric hindrance during transition to the latent state.

When compared with human PAI-1, 5-fold slower latency transition of glycosylated zfPAI-1 has been demonstrated. When fish PAI-1 compared with human PAI-1, a single N-linked glycan at Asn^185^ in the gate region was detected (RCL passes through this region in the period of latency transition).

It is known that deglycosylation has no effect during the latency transition of human PAI-1; on the other hand, 50-fold faster latency transition was observed for deglycosylated zebrafish PAI-1 (zfPAI-1) in contrast to the glycosylated zfPAI-1. Moreover, deglycosylated zebrafish PAI-1 (zfPAI-1) is about 25-fold faster than nonglycosylated human PAI-1. Presence of an N-linked glycan in the gate region and absence of glycan-induced structural changes were confirmed when glycosylated fish PAI-1 was analyzed based on X-ray crystal structure [136].

Investigations on insulin-resistant old rats showed that the high degree of PAI-1 glycosylation and activity related to an increased cardiovascular risk associated with insulin-resistant states [135]. Serrano et al. reported that glycosylation determined a 10-fold higher specific activity against u-PA and 2.3 against t-PA inhibition [135]. Thus, highly glycosylated PAI-1 form can implicate higher concentrations of active PAI-1 and an increased cardiovascular risk in insulin-resistant old rats.

On the other hand, there is increasing evidence that insulin resistance abdominal obesity increases PAI-1 antigen and activity levels. The likely mechanisms may involve upregulation of PAI-1 synthesis by insulin, glucocorticoids, angiotensin II, fatty acids, and cytokines such as tumour necrosis factor-alpha and transforming growth factor-beta. PAI-1 glycosylation is another potential target to modulate the enhanced effects of PAI-1 in these patients [82, 137].

## 8. Future Prospects

The influence of PAI-1 on the pathophysiology of complex diseases may depend on genetic and environmental effects and their interactions. Furthermore, the mechanisms may differ across the populations. Rather than single-site allelic and genotypic associations, multilocus genotype equilibrium and multilocus genotype and environmental risk factor associations using bioinformatic methods might be necessary to investigate the effects of PAI-1 on disease mechanisms. Enhanced stability of PAI-1 contributes to the pathophysiology of a wide range of complex diseases including atherosclerosis, dementia, and cancer.

A number of potential mechanisms may be associated with the increased functional stability of PAI-1 including point mutation(s) in the coding domain sequences (CDSs) of PAI-1 gene, binding stabilizing cofactors, and posttranslational modifications in the PAI-1 protein. Systemic or local treatment with PAI-1 inhibitors may offer potential treatment alternatives to the near orphan status for novel drug development.

Moreover, PAI-1 is a potential biological marker that can be progressively considered in the prognostic evaluation and disease monitoring and as a treatment target of age-related conditions in the future.

## Figures and Tables

**Table 1:** Random mutagenesis approach to the stability of PAI-1.

Mutation	Stability increase	*t*_1/2_	Reference
I91L	9-fold	18 h	[61]
N150H, K154T, Q319L, and M354I	72-fold	145 h	[125]
N150H, K154T, Q319L, M354I, and Q301P	75-fold	150 h	[126]
Combination of mutations at positions 50, 56, 61, 70, 94, 150, 222, 223, 264, and 331	122-fold	245 h	[127]
Disulfide mutant	350-fold	700 h	[128]
